# A Novel Image Encryption Scheme Based on Self-Synchronous Chaotic Stream Cipher and Wavelet Transform

**DOI:** 10.3390/e20060445

**Published:** 2018-06-06

**Authors:** Chunlei Fan, Qun Ding

**Affiliations:** Electrical Engineering College, Heilongjiang University, Harbin 150080, China

**Keywords:** hyperchaotic system, self-synchronous stream cipher, permutation entropy, image encryption, wavelet transform

## Abstract

In this paper, a novel image encryption scheme is proposed for the secure transmission of image data. A self-synchronous chaotic stream cipher is designed with the purpose of resisting active attack and ensures the limited error propagation of image data. Two-dimensional discrete wavelet transform and Arnold mapping are used to scramble the pixel value of the original image. A four-dimensional hyperchaotic system with four positive Lyapunov exponents serve as the chaotic sequence generator of the self-synchronous stream cipher in order to enhance the security and complexity of the image encryption system. Finally, the simulation experiment results show that this image encryption scheme is both reliable and secure.

## 1. Introduction

With the rapid development of social networking, cloud computing, and mobile network communication technology, the problem of secure storage and real-time transmission of image data is increasingly important. Encryption and digital watermarking technology play an important role in guaranteeing the security of multimedia data [1]. However, because of the high correlation and redundancy of adjacent pixels of the digital image, some international standard encryption algorithms are not suitable for image encryption, including 3DES (Triple Data Encryption Algorithm), IDEA (International Data Encryption Algorithm), and AES (Advanced Encryption Standard), etc. On the other hand, the chaotic nonlinear dynamic system has some good characteristics, such as positive Lyapunov exponents, ergodicity, sensitivity to initial conditions, topological transitivity, and unpredictability [2,3,4,5], and was widely applied in the field of cryptography and secret communication. In recent years, in order to better solve the security transmission of digital images, some scholars have put forward a series of image security encryption schemes based on the chaotic system and the inherent characteristics of digital images [6,7,8,9]. For example, Ping et al. [10] proposed a permutation-substitution image encryption scheme with the Henon map, which can resist a chosen-plaintext attack and known-plaintext attack. Ye et al. [11] put forward an efficient symmetric image encryption algorithm based on an intertwining Logistic map. Haroun [12] came up with a real-time image encryption scheme using a low-complexity discrete 3D dual chaotic cipher.

However, these image encryption schemes generally use low-dimensional chaotic systems or high-dimensional chaotic systems with only one positive Lyapunov exponent such as Logistic, Tent, Henon, and Lorenz, etc. Compared with the high-dimensional hyperchaotic system with more than two positive Lyapunov exponents, the complexity of nonlinear dynamic characteristics of the above chaotic systems are lower. Additionally, because of the influence of the calculation precision and the quantization method, the chaotic binary sequences generated by the low-dimensional chaotic systems emerge with short periodic phenomena [13,14], which will seriously affect the security of image encryption. Furthermore, the above image encryption schemes usually adopt the synchronous sequence cipher based on the chaotic binary sequences [15,16]. In synchronous stream ciphers, the key stream is independent of plaintext or ciphertext. In the process of communication, the sender and receiver must keep accurate synchronization. If the synchronization mechanism is broken by active attack, the receiver will not be able to decrypt the ciphertext correctly. For instance, if an attacker inserts or removes a certain number of bits ciphertext, it will immediately destroy the synchronization mechanism of the synchronous sequence cipher. Therefore, this encryption method cannot resist active attack [17]. On the basis of the above image encryption problem, we proposed a novel image encryption scheme based on self-synchronous chaotic stream cipher and wavelet transform. Firstly, a two-dimensional discrete wavelet transform is used to convert the original image from the spatial domain to the frequency domain with the purpose of strengthening the difficulty of cracking. Secondly, the pixel value of the image is scrambled by Arnold mapping. Finally, the scrambled image is encrypted by self-synchronous chaotic stream cipher. This algorithm uses a four-dimensional hyperchaotic system with four positive Lyapunov exponents and a self-synchronous stream cipher mechanism. The generation of the key stream of the self-synchronous stream cipher is not independent of the plaintext and ciphertext stream but is related to the seed key and *n*-bits ciphertext that have been generated before. In the process of ciphertext transmission, the 1-bit ciphertext error will only affect the correct decryption of the *n*-bits ciphertext in the back. The decryption process returns to normal after this time. Therefore, this scheme cannot only resist active attack but also ensures the limited error propagation of image data. The experimental results show that the encryption scheme has good security.

The rest of this paper is organized as follows: Section 2 introduces a four-dimensional hyperchaotic system and the design scheme of the self-synchronous chaotic stream cipher. Furthermore, the performance of the discrete chaotic sequence was analyzed by multi-scale permutation entropy and NIST-800-22 test. In Section 3, a novel image encryption scheme is proposed and a detailed security analysis is carried out with histogram and information entropy analyses, etc. Section 4 summarizes the conclusion of this paper.

## 2. Design and Implementation of Self-Synchronous Chaotic Stream Cipher

### 2.1. The Description of Four-Dimensional Discrete Chaotic System

In this section, a four-dimensional chaotic system is constructed through the Chen–Lai algorithm [18,19]. The discrete dynamic equations of the system can be expressed as the follows:

(1) $$\left(\begin{array}{c} x_{1}(k+1)\\ x_{2}(k+1)\\ x_{3}(k+1)\\ x_{4}(k+1) \end{array}\right)=A\left(\begin{array}{c} x_{1}(k)\\ x_{2}(k)\\ x_{3}(k)\\ x_{4}(k) \end{array}\right)+({\left\|A\right\|}_{2}+e^{c})\left(\begin{array}{c} x_{1}(k)\\ x_{2}(k)\\ x_{3}(k)\\ x_{4}(k) \end{array}\right)(\mathrm{mod}1)$$

where ${\left\|\cdot \right\|}_{2}$ and e represent Euclidean norm and mathematical constant, respectively. The mod is the module operations, and c is control parameter. Furthermore, matrix A is given as follows:

(2) $$A=\left(\begin{array}{cccc} A_{11} & A_{12} & A_{13} & A_{14}\\ A_{21} & A_{22} & A_{23} & A_{24}\\ A_{31} & A_{32} & A_{33} & A_{34}\\ A_{41} & A_{42} & A_{43} & A_{44} \end{array}\right)=\left(\begin{array}{cccc} 0.7 & 0.4 & 0.1 & 0.2\\ 0.2 & -0.5 & 0.1 & 0\\ 0 & -1/3 & 0.1 & 0\\ 0 & -1/4 & 0.3 & 0.6 \end{array}\right).$$

**Figure 1:**
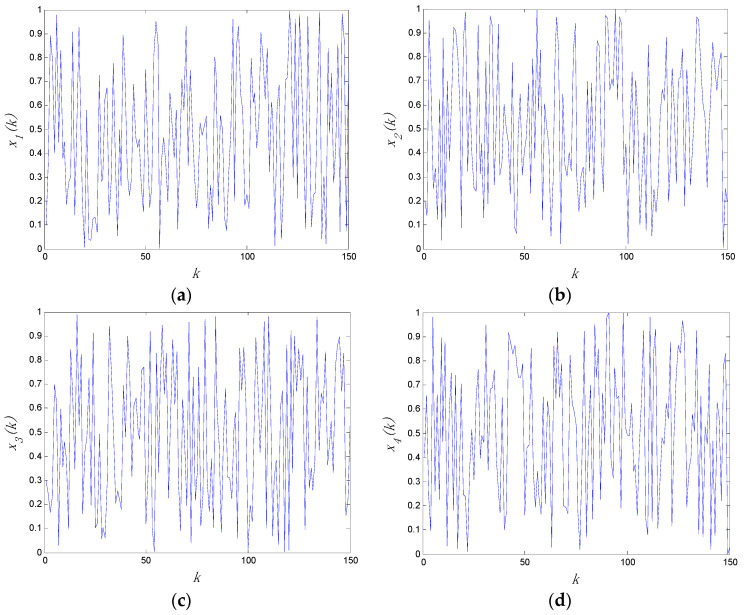
The chaotic time series of the four-dimensional hyperchaotic system with: (**a**) $x_{1}(k)$; (**b**) $x_{2}(k)$; (**c**) $x_{3}(k)$; (**d**) $x_{4}(k)$.

When c=3, the Lyapunov exponents of the system are given by $LE_{1}=3.0128$, $LE_{2}=3.0454$, $LE_{3}=3.0717$, and $LE_{4}=3.0799$. The number of positive Lyapunov exponents are more than two. Thus, the system is a four-dimensional hyperchaotic system. The chaotic time series of the four-dimensional hyperchaotic system are shown in Figure 1.

### 2.2. Quantization and Performance Analysis of Discrete Chaotic Sequences

#### 2.2.1. Binary Quantization Method

For the above chaotic system, $x_{j}(k)\in (0,1)$ with j=1,2,3,4. In this paper, we adopt the binary quantization method to quantize discrete chaotic real value sequences. The corresponding quantization method is defined as follows:

(3) $$\begin{array}{cc} Q_{j}(k)=\{\begin{array}{c} \begin{array}{cc} 0 & x_{j}(k)<t_{d} \end{array}\\ \begin{array}{cc} 1 & x_{j}(k)\geq t_{d} \end{array} \end{array} & j=1,2,3,4 \end{array}$$

where $Q_{j}(k)$ is the quantized chaotic binary sequence, and $t_{d}$ represents the quantization threshold with $t_{d}\;=\;0.5$.

#### 2.2.2. Multi-Scale Permutation Entropy Analysis

Multi-scale permutation entropy (MPE) [20,21] has the advantages of high robustness and fast computational speed. It is widely applied in the measurement of binary sequence complexity and nonlinear system analysis. In this section, we perform a multi-scale permutation entropy analysis for the above chaotic binary sequence. The parameters of MPE have embedding dimension m, delay factor τ, and scale factor s. For the choice of the parameter values of the multi-scale permutation entropy with the purpose of calculating the complexity of chaotic binary sequences, Sun et al. [22] and Xu et al. [23] give the recommended parameter range in order to obtain more accurate entropy values. On the basis of the theoretical research of the above references, in this experiment, we set m = 3, τ = 2, and s∈[3,7], respectively. The experimental results are shown in Table 1. As can be seen from Table 1, all MPE values of chaotic binary sequences are more than 0.9 and display good sequence complexity.

#### 2.2.3. NIST-800-22 Test

NIST-800-22 is a statistical test suite for random and pseudorandom number generators for cryptographic applications. This test standard was enacted by the National Institute of Standards and Technology (NIST). The test statistic is used to calculate a *p*-value that summarizes the strength of the evidence against the null hypothesis. On the basis of the results of NIST test, we can judge whether or not this chaotic binary sequence is suitable for a cryptographic algorithm. NIST-800-22 is made up of 16 test methods, including the longest run test, cumulative sums, and the linear complexity test, etc. For these tests, each *p*-value is the probability that a perfect random number generator would have produced a sequence less random than the sequence that was tested, given the kind of non-randomness assessed by the test. A significance level (α) can be chosen for the tests. If p-value≥α, then the null hypothesis is accepted; i.e., the sequence appears to be random. If p-value<α, then the null hypothesis is rejected; i.e., the sequence appears to be non-random. Typically, α is chosen in the range [0.001,0.01]. Common values of α in cryptography are about 0.01 based on the NIST-800-22 test standard [24]. The experimental results of NIST-800-22 test are shown in Table 2. Table 2 shows that the chaotic binary sequences $Q_{1}(k)$, $Q_{2}(k)$, $Q_{3}(k)$, and $Q_{4}(k)$ passed all the tests. These sequences show good randomness and meet the requirements of the stream cipher.

### 2.3. The Design of Self-Synchronous Chaotic Stream Cipher

The self-synchronous stream cipher is also known as the asynchronous stream cipher. The generation of the key stream of self-synchronous stream cipher is not independent of the plaintext and ciphertext stream but is related to the seed key and some ciphertext that has been generated before.

**Figure 2:**
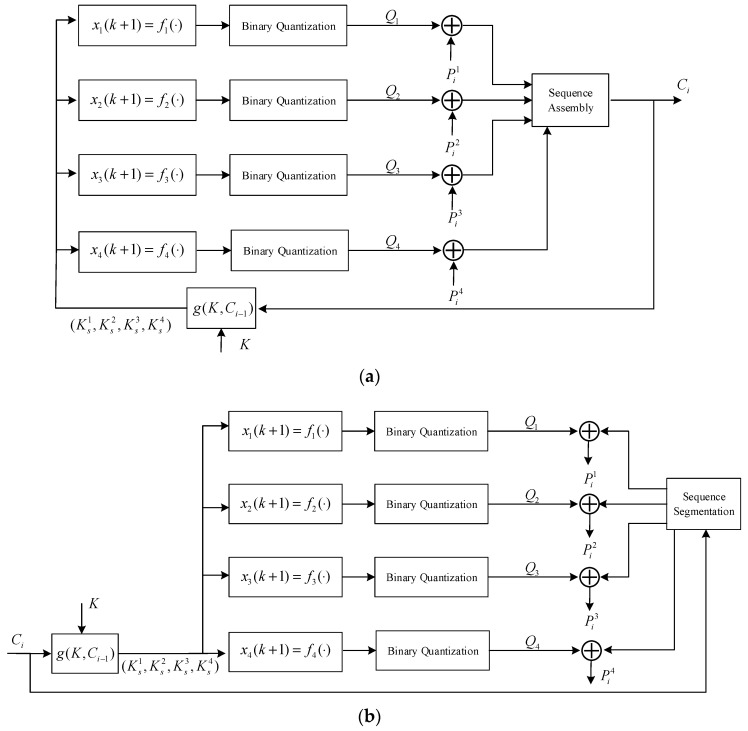
The self-synchronous chaotic stream cipher with: (**a**) decryption block diagram; (**b**) decryption block diagram.

It has the advantages of limited error propagation, self-synchronous and ciphertext statistical diffusion. In this section, the encryption and decryption block diagram of the self-synchronous stream cipher based on the four-dimensional hyperchaotic system is shown in Figure 2.

Where K, $\left(K_{s}^{1},K_{s}^{2},K_{s}^{3},K_{s}^{4}\right)$, ⊕ and function g(⋅) represent the seed key, subkey, exclusive OR (XOR) operational character and subkey generation function, respectively. $C_{i-1}$ and $C_{i}$ are the ciphertext stream generated at two adjacent moments. $Q_{1}$, $Q_{2}$, $Q_{3}$, and $Q_{4}$ are the quantized chaotic binary sequence. $P_{i}^{1}$, $P_{i}^{2}$, $P_{i}^{3}$, and $P_{i}^{4}$ are the adjacent plaintext stream with the purpose of parallel encryption. According to Equations (1) and (2), $f_{1}(\cdot )$, $f_{2}(\cdot )$, $f_{3}(\cdot )$, and $f_{4}(\cdot )$ can be given as Equation (4).

(4) $$\{\begin{array}{c} x_{1}(k+1)=f_{1}(\cdot )=A_{11}x_{1}(k)+A_{12}x_{2}(k)+A_{13}x_{3}(k)+A_{14}x_{4}(k)+({\left\|A\right\|}_{2}+{\text{e}}^{c})x_{1}(k)(\mathrm{mod}1)\\ x_{\text{2}}(k+1)=f_{\text{2}}(\cdot )=A_{\text{2}1}x_{1}(k)+A_{\text{2}2}x_{2}(k)+A_{\text{2}3}x_{3}(k)+A_{\text{2}4}x_{4}(k)+({\left\|A\right\|}_{2}+{\text{e}}^{c})x_{\text{2}}(k)(\mathrm{mod}1)\\ x_{\text{3}}(k+1)=f_{\text{3}}(\cdot )=A_{\text{3}1}x_{1}(k)+A_{\text{3}2}x_{2}(k)+A_{\text{3}3}x_{3}(k)+A_{\text{3}4}x_{4}(k)+({\left\|A\right\|}_{2}+{\text{e}}^{c})x_{\text{3}}(k)(\mathrm{mod}1)\\ x_{4}(k+1)=f_{4}(\cdot )=A_{41}x_{1}(k)+A_{42}x_{2}(k)+A_{43}x_{3}(k)+A_{44}x_{4}(k)+({\left\|A\right\|}_{2}+{\text{e}}^{c})x_{4}(k)(\mathrm{mod}1) \end{array}$$

The encryption process of the self-synchronous chaotic stream cipher can be described as follows:

1. The subkey $\left(K_{s}^{1},K_{s}^{2},K_{s}^{3},K_{s}^{4}\right)$ is generated by function g(⋅) and $K_{s}^{j}\in (0,1)$ with j=1,2,3,4. Where K and $C_{i-1}$ are the 0-1 binary sequence with length of 32 bits. The function g(⋅) is given as follows:
  (5) $$\{\begin{array}{c} K=(k_{1},k_{2},\cdots ,k_{32})\\ C_{i-1}=(c_{i-1}^{1},c_{i-1}^{2},\cdots ,c_{i-1}^{32})\\ K_{s}^{1}=g(K,C_{i-1})=\sum_{v=1}^{8}\left(k_{4v-3}⊕c_{i-1}^{4v-3}\right)2^{-v}\\ K_{s}^{2}=g(K,C_{i-1})=\sum_{v=1}^{8}\left(k_{4v-2}⊕c_{i-1}^{4v-2}\right)2^{-v}\\ K_{s}^{3}=g(K,C_{i-1})=\sum_{v=1}^{8}\left(k_{4v-1}⊕c_{i-1}^{4v-1}\right)2^{-v}\\ K_{s}^{4}=g(K,C_{i-1})=\sum_{v=1}^{8}\left(k_{4v}⊕c_{i-1}^{4v}\right)2^{-v} \end{array}$$
2. The generated subkey $\left(K_{s}^{1},K_{s}^{2},K_{s}^{3},K_{s}^{4}\right)$ is used as the state variable $\left(x_{1}(k),x_{2}(k),x_{3}(k),x_{4}(k)\right)$ of the four-dimensional hyperchaotic system. The key stream $Q_{1}$, $Q_{2}$, $Q_{3}$ and, $Q_{4}$ with length of 8 bits are generated by hyperchaotic Equation (4) and a binary quantization operation with the purpose of parallel encryption.
3. Ciphertext $C_{i}$ is generated by the Equation (6). At the same time, the $C_{i}$ will feedback to the function g(⋅) with the purpose of generating the next round subkey $\left(K_{s}^{1},K_{s}^{2},K_{s}^{3},K_{s}^{4}\right)$. Where || is sequence assembly operation.
  (6) $$\{\begin{array}{c} Q_{1}⊕P_{i}^{1}=c_{i}^{1},c_{i}^{2},\cdots ,c_{i}^{8}\\ Q_{2}⊕P_{i}^{2}=c_{i}^{9},c_{i}^{10},\cdots ,c_{i}^{16}\\ Q_{3}⊕P_{i}^{3}=c_{i}^{17},c_{i}^{18},\cdots ,c_{i}^{24}\\ Q_{4}⊕P_{i}^{4}=c_{i}^{25},c_{i}^{26},\cdots ,c_{i}^{32}\\ C_{i}=(Q_{1}⊕P_{i}^{1})||(Q_{2}⊕P_{i}^{2})||(Q_{3}⊕P_{i}^{3})||(Q_{4}⊕P_{i}^{4})=c_{i}^{1},c_{i}^{2},\cdots ,c_{i}^{32} \end{array}$$

The decryption process of the self-synchronous chaotic stream cipher is similar to the encryption process, which is not repeated here.

## 3. A Novel Image Encryption Scheme Based on Self-Synchronous Chaotic Stream Cipher

### 3.1. The Description of the Image Encryption Scheme

**Figure 3:**
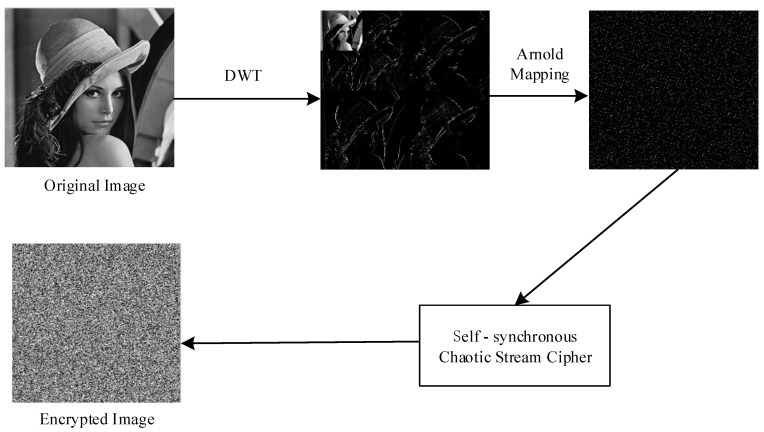
The encryption process of the proposed scheme.

In this section, a novel image encryption scheme is proposed based on self-synchronous chaotic stream cipher, Arnold mapping, and two-dimensional discrete wavelet transform (DWT). The encryption process of the proposed scheme is shown in Figure 3. Firstly, the spatial domain of the image is transformed into the frequency domain by two-layer DWT. Secondly, Arnold mapping is implemented with the purpose of obtaining good diffusion effectiveness. Finally, on the basis of self-synchronous chaotic stream cipher, the scrambled image is encrypted to ensure its security.

#### 3.1.1. Discrete Wavelet Transform

**Figure 4:**
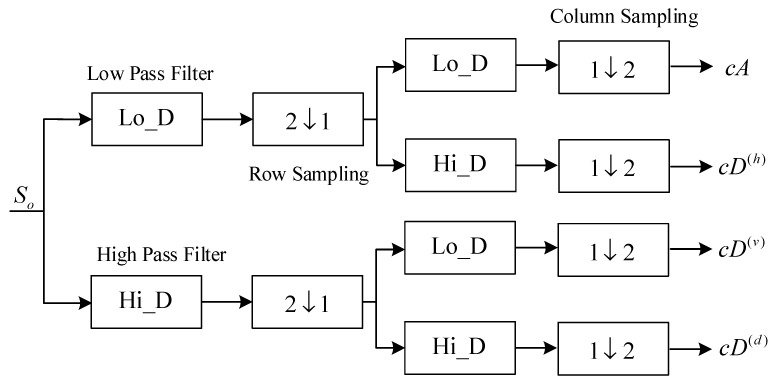
The principle block diagram of the two-dimensional discrete wavelet transform.

The DWT plays an important role in image compression and image information processing. The decomposition process of signal $S_{o}$ by the two-dimensional DWT is shown in Figure 4. A two-dimensional matrix $S_{o}$ can be decomposed into four groups of coefficients $\left[cA,cD^{\left(h\right)},cD^{\left(v\right)},cD^{\left(d\right)}\right]$. Where cA, $cD^{\left(h\right)}$, $cD^{\left(v\right)}$, and $cD^{\left(d\right)}$ represent approximate coefficient (low frequency component), horizontal detail coefficient, vertical detail coefficient, and diagonal detail coefficients, respectively. Furthermore, the approximate coefficient cA can continue to be decomposed by the same method. On the basis of the above image encryption scheme, in order to obtain the transformation coefficient, the pixel value of the grayscale image is processed by two-dimensional discrete wavelet transform. Thereafter, the spatial domain of the digital image is transformed into the frequency domain with the purpose of enhancing the pixel scrambling effect.

#### 3.1.2. Arnold Mapping

Arnold mapping [25], also known as cat mapping, is a chaotic mapping method for repeated folding and stretching transformation in a limited area. It is widely applied to pixel scrambling of images. The mathematical equation of Arnold mapping is given as follows:

(7) $$\left[\begin{array}{c} X_{n+1}\\ Y_{n+1} \end{array}\right]=\left[\begin{array}{cc} 1 & a\\ b & ab+1 \end{array}\right]\left[\begin{array}{c} X_{n}\\ Y_{n} \end{array}\right](\mathrm{mod}M)$$

where $\left(X_{n},Y_{n}\right)$ and $\left(X_{n+1},Y_{n+1}\right)$ represent the pixel coordinates of the original image and the pixel coordinates of transformed image, respectively. In addition, $X_{n}$, $Y_{n}$, $X_{n+1}$, $Y_{n+1}\in \{0,1,\cdots ,N-1\}$, and a=1, b=1. The variable M is the size of the image.

### 3.2. Security Analysis of Image Encryption Scheme

In this section, the Lena, fruits, and airplane gray images with a size of 256 × 256 are encrypted using the above image encryption scheme based on self-synchronous chaotic stream cipher. The security analysis results of image encryption are shown below.

#### 3.2.1. Histogram Analysis

**Figure 5:**
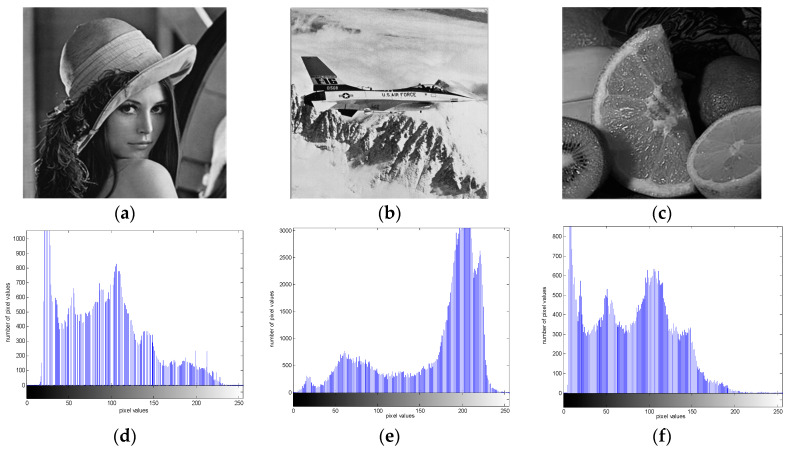

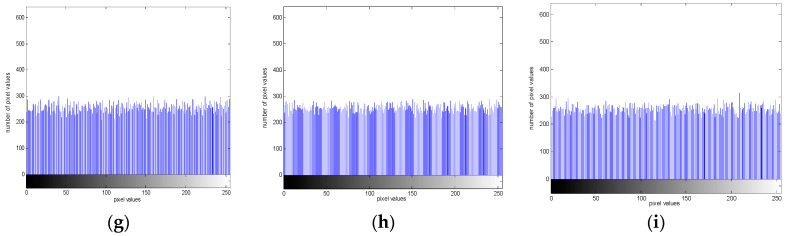
Histogram test with: (**a**) plain-image of Lena; (**b**) plain-image of airplane; (**c**) plain-image of fruits; (**d**) histogram of plain-image of Lena; (**e**) histogram of plain-image of airplane; (**f**) histogram of plain-image of fruits; (**g**) histogram of encrypted image of Lena; (**h**) histogram of encrypted image of airplane; (**i**) histogram of encrypted image of fruits.

The histogram of the image is an important statistical feature of the image. It can be considered as the gray density function of the image. One of the evaluation criteria of image encryption effect is whether the gray histogram of the ciphertext image has the characteristics of uniform distribution. The results of the grayscale histogram are shown in Figure 5, and the horizontal and vertical coordinates of the gray histogram represent the pixel values and number of pixel values, respectively. As can be seen from the Figure 5g–i, the pixel values of the ciphertext images are so evenly distributed that it is difficult for the attacker to extract the plaintext pixel statistical characteristics from the ciphertext. Therefore, this image encryption scheme can resist statistical attacks well.

#### 3.2.2. Correlation Analysis of Adjacent Pixels

Correlation analysis refers to the analysis of two variables with correlation so as to measure the correlation degree of two variables. The correlation of adjacent pixels can reflect the scrambling effect of image pixels. The mathematical equation for the correlation of adjacent pixels is shown as follows [26]:

(8) $$E(x)=\frac{1}{N}\sum_{k=1}^{N}x_{k}$$

(9) $$D(x)=\frac{1}{N}\sum_{k=1}^{N}{\left(x_{k}-E(x)\right)}^{2}$$

(10) $$\mathrm{cov}(x,y)=\frac{1}{N}\sum_{k=1}^{N}\left(x_{k}-E(x))(y_{k}-E(y)\right)$$

(11) $$\rho_{xy}=\frac{\mathrm{cov}(x,y)}{\sqrt{D(x)}\sqrt{D(y)}}$$

where $x_{k}$ and $y_{k}$ represent the grey values of two adjacent pixels, and N is the number of randomly selected adjacent pixels from the original or encrypted image. The $\rho_{xy}$, E(x), D(x) and cov(x,y) represent the correlation coefficient, mean value, variance, and covariance, respectively.

**Figure 6:**
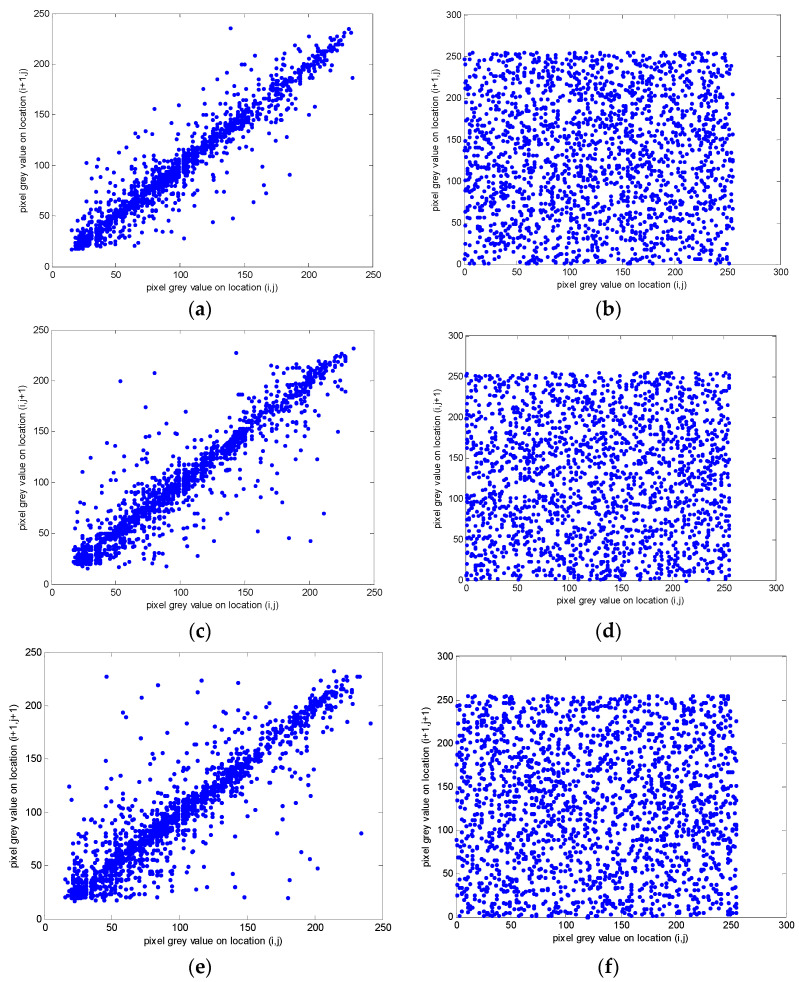
The correlation plots of two adjacent pixels for the plain and encrypted images of Lena with: (**a**) horizontal correlation of plain-image of Lena; (**b**) horizontal correlation of cipher-image of Lena; (**c**) vertical correlation of plain-image of Lena; (**d**) vertical correlation of cipher-image of Lena; (**e**) diagonal correlation of plain-image of Lena; (**f**) diagonal correlation of cipher-image of Lena.

For the correlation analysis experiment, we randomly selected 2000 pairs of adjacent pixels in horizontal, vertical, and diagonal directions from the plain and encrypted images of Lena. The experimental results are shown in Figure 6. Where (i,j) represents the position coordinates of this pixel in the image. As can be seen from the figure, the correlation of cipher-images is much lower than that of plain-images. Furthermore, Table 3 shows the correlation analysis of the adjacent pixels with the Lena, airplane, and fruits image. Obviously, the correlation coefficient of the plain-image is close to 1. On the contrary, the correlation coefficient of the cipher-image is close to 0, which indicates a good performance.

#### 3.2.3. Peak Signal-To-Noise Ratio (PSNR) Analysis

The peak signal-to-noise ratio is an objective criterion for evaluating images, and its mathematical equation is given as follows:

(12) $$\mathrm{PSNR}=10\log_{10}(L^{2}/\mathrm{MSE})$$

(13) $$\mathrm{MSE}=\frac{1}{M^{2}}\sum_{i=1}^{M}\sum_{j=1}^{M}{\left(I^{\prime }(i,j)-I(i,j)\right)}^{2}$$

where M × M is the size of image, $I^{\prime }(i,j)$, and I(i,j) represent the pixel value of encrypted and original images, respectively. MSE is mean squared error, and L is the range of gray values in the image. Generally speaking, the better the encryption effect is, the smaller the PSNR of the image becomes. The results of the PSNR test is shown in Table 4. For the test result of our scheme, it is the average PSNR of the Lena, airplane, and fruits images. Similarly, for the image encryption method of Yin et al. [27] and Zhu [28], this PSNR represents the average value of multiple images. As can be seen from the table, our scheme has a smaller PSNR value, which shows a good encryption effect.

#### 3.2.4. Information Entropy Analysis

Information entropy can measure the distribution of gray values in images. The more random the gray value distribution, the greater the information entropy of the image. According to the information theory of Shannon, the information entropy can be defined as follows:

(14) $$H(s)=-\sum_{i=0}^{2^{n}-1}P(s_{i})\log_{2}(P(s_{i}))$$

where $P(s_{i})$ represents the probability of symbol $s_{i}$, n is the number of bits required to store each pixel value, $2^{n}$ is the total states of the information source s. When n = 8, the theoretical value of information entropy is 8. The information entropy test was performed for the encrypted images of Lena, airplane, and fruits. Table 5 shows the experimental results of the entropy test. The test result of our scheme is the average entropy value of the Lena, airplane, and fruits images. For the scheme of Liu et al. [29], Liu et al. [30], and Niyat [31], these entropy values represent the average value of multiple images. As can be seen from the table, the entropy value of encrypted image of our scheme is closer to the theoretical value 8. Therefore, this scheme can effectively resist the information entropy attack.

## 4. Discussion

In this paper, we proposed a novel image encryption scheme with the purpose of ensuring secure transmission of image data. A four-dimensional hyperchaotic system is constructed to act as the chaotic sequence generator. Moreover, the performance of chaotic binary sequences is analyzed by multi-scale permutation entropy and the NIST-800-22 test. The test results show that the binary sequence has good randomicity and security. On the basis of the chaotic sequence generator, a self-synchronous stream cipher is designed to encrypt the image data. The generation of the key stream of the chaotic stream cipher is related to the seed key and a certain number of bits ciphertext that has been generated previously. It has the advantages of limited error propagation, self-synchronous and ciphertext statistical diffusion, which can satisfy the security and stability of image encryption system. Finally, a novel image encryption scheme is designed based on the self-synchronous stream cipher. Arnold mapping and wavelet transform are used to obtain a good scrambling effect for the digital image. Some simulation results show that the proposed scheme is both reliable and secure. 

## Figures and Tables

**Table 1:** The multi-scale permutation entropy (MPE) value of chaotic binary sequences with $Q_{1}(k)$, $Q_{2}(k)$, $Q_{3}(k)$, and $Q_{4}(k)$.

Scale Factor s	$Q_{1}(k)$	$Q_{2}(k)$	$Q_{3}(k)$	$Q_{4}(k)$
3	0.9201	0.9260	0.9177	0.9132
4	0.9366	0.9382	0.9449	0.9410
5	0.9548	0.9488	0.9567	0.9377
6	0.9590	0.9572	0.9585	0.9526
7	0.9552	0.9533	0.9704	0.9553

**Table 2:** NIST-800-22 test of chaotic binary sequences.

Test Item	$Q_{1}(k)$	$Q_{2}(k)$	$Q_{3}(k)$	$Q_{4}(k)$	Result
	p-Value	p-Value	p-Value	p-Value	
Approximate Entropy	0.026853	0.013829	0.068205	0.034937	Success
Block Frequency	0.058378	0.870831	0.724584	0.297646	Success
Cumulative Sums	0.459642	0.069717	0.963210	0.328997	Success
FFT	0.358795	0.919848	0.081236	0.713570	Success
Frequency	0.435391	0.447255	0.888660	0.193601	Success
Linear Complexity	0.186537	0.203633	0.569565	0.232544	Success
Longest Run	0.359643	0.087189	0.789913	0.250387	Success
Non-Overlapping Template	0.348045	0.680967	0.106169	0.068529	Success
Overlapping Template	0.512834	0.063236	0.020689	0.490518	Success
Random Excursions	0.319514	0.181174	0.524622	0.304589	Success
Random Excursions Variant	0.579380	0.177934	0.108254	0.659874	Success
Rank	0.949536	0.648387	0.862457	0.648387	Success
Runs	0.340097	0.086469	0.041369	0.027231	Success
Serial Test-1	0.407933	0.213432	0.648688	0.814738	Success
Serial Test-2	0.462490	0.880617	0.584615	0.512974	Success
Maurer’s Universal	0.026152	0.538143	0.142680	0.600293	Success

**Table 3:** Correlation analysis of adjacent pixels for the Lena, airplane, and fruits images.

Direction	Horizontal	Vertical	Diagonal
Plain-image of Lena	0.9577	0.9440	0.9126
Cipher-image of Lena	−0.0082	0.0027	0.0030
Plain-image of Airplane	0.9147	0.9225	0.9109
Cipher-image of Airplane	0.0334	−0.0285	−0.0073
Plain-image of Fruits	0.9540	0.9497	0.9459
Cipher-image of Fruits	−0.0273	−0.0176	−0.0026

**Table 4:** Peak signal-to-noise ratio (PSNR) test with different methods.

Methods	PSNR Value
Our scheme	8.1543
Yin et al. [27]	8.4100
Zhu [28]	9.2322

**Table 5:** Information entropy test with different methods.

Methods	Entropy Value
Our scheme	7.9971
Liu et al. [29]	7.9914
Liu et al. [30]	7.9851
Niyat et al. [31]	7.9877
